# Loop-mediated isothermal amplification assays for the rapid discrimination of *Treponema pallidum* lineages and subspecies

**DOI:** 10.1128/spectrum.01565-25

**Published:** 2025-10-30

**Authors:** Kazuo Imai, Yuki Ohama, Akihiro Sato, Masashi Tanaka, Ryuha Omachi, Keita Takeuchi, Hitomi Mizushina, Shu-ichi Nakayama, Takuya Maeda, Yukihiro Akeda

**Affiliations:** 1 Department of Clinical Laboratory Medicine, Saitama Medical University 681431, Saitama, Japan; 2 KARADA Internal Medicine Clinic, Tokyo, Japan; 3 Department of Bacteriology I, National Institute of Infectious Diseases, Japan Institute for Health Security 13511, Tokyo, Japan; Petrified Bugs LLC, Miami, Florida, USA

**Keywords:** syphilis, *Treponema*, LAMP, MLST, genotyping

## Abstract

Molecular surveillance aimed at identifying the subspecies and lineages of *Treponema pallidum* is important. Conventional molecular methods for genotyping are effective but are also time-consuming and costly. This study aimed to develop and evaluate a novel loop-mediated isothermal amplification (LAMP) method using quenching probes to quickly distinguish *T. pallidum* subsp. *pallidum* lineages (Nichols, SS14Ω-A, SS14Ω-B 1A, and SS14-East Asia) and *T. pallidum* subsp. *endemicum* circulating in East Asia. We developed novel LAMP assays for genotyping the *tp0705* (amino acid variants of 506, 625, and 708) and *tp0548* genes (a 9 bp insertion region). The LAMP assays were used to genotype 133 clinical samples (Nichols, *n* = 9; SS14Ω-A, *n* = 7; SS14Ω-B 1A, *n* = 2; SS14-East Asia, *n* = 110; and *T. pallidum* subsp. *endemicum*, *n* = 5) collected from adult patients diagnosed with syphilis in Japan. The genotyping results of the LAMP assays were compared with the results from Sanger or whole-genome sequencing. The lower detection limit of the LAMP assays for *tp0705* and *tp0548* genotyping was 20 DNA copies/reaction. Of the 133 samples analyzed, the genotypes, lineages, and subspecies of *T. pallidum* were identified in 129 (97.0%) samples by the LAMP assays. The genotyping results of the LAMP assays were completely consistent with those of Sanger and whole-genome sequencing. Our novel LAMP genotyping assays can rapidly and easily discriminate the lineages and subspecies of *T. pallidum* circulating in East Asia. This method will contribute to the epidemiological surveillance of *T. pallidum* in East Asia.

**IMPORTANCE:**

Syphilis remains a significant global public health concern. Molecular surveillance is essential for understanding the transmission dynamics and spread of various *Treponema pallidum* subsp. *pallidum* lineages, including emerging strains such as *Treponema pallidum* subsp. *endemicum* (TEN). However, conventional genotyping methods like multi-locus sequence typing and whole-genome sequencing are costly, time-consuming, and require specialized equipment and expertise. In this study, we developed a novel loop-mediated isothermal amplification assay with quenching probes that enables rapid, accurate, and low-cost identification of major *T. pallidum* lineages and subspecies. Our method demonstrated high sensitivity and specificity using clinical specimens and successfully distinguished among Nichols, SS14 variants, and TEN. This assay is well suited for large-scale epidemiological studies and may support more effective and timely public health responses to the rising incidence of syphilis in East Asia and beyond.

## INTRODUCTION

Syphilis, a sexually transmitted disease, is a global public health concern. The causative agent of syphilis is the spirochete *Treponema pallidum* subsp. *pallidum* (TPA). The major transmission route is sexual contact, but mother-to-fetus transmission can occur *in utero* via the transplacental route and during delivery via contact transmission from a lesion. In the past few decades, the number of newly diagnosed cases of syphilis has increased significantly in Japan and several other countries, leading to increases in fetal loss, stillbirth, neonatal death, and congenital infection (1–3). Syphilis often co-occurs with HIV, other sexually transmitted infections, and emerging infections such as mpox (4, 5). The vigorous and sustained molecular surveillance of TPA is conducted to enhance clinical care, prevention, and control efforts by contributing to a better understanding of TPA acquisition and transmission. According to genetic analysis, TPA is separated into two genetically distinct groups: the Nichols and SS14 lineages. Whole-genome sequencing revealed that the SS14 lineage can be partitioned into two lineages: SS14Ω-A and SS14Ω-B (6–8). Furthermore, SS14Ω-B can be partitioned into two sub-lineages: SS14Ω-B 1A (spread in North America/Europe) and 1B (spread in East Asia) (6–8). In addition, *Treponema pallidum* subsp. *endemicum* (TEN), which has long been endemic in the Middle East and causes endemic syphilis (bejel), has been detected in several patients with syphilis-like symptoms outside of the typical endemic countries such as Cuba (9, 10), France (11), and Japan (12). In East Asia, molecular surveillance data show that the dominant syphilis-causing *Treponema* belongs to the SS14Ω-B 1B (SS14-East Asia) lineage, followed by Nichols, SS14Ω-A, TEN, and SS14Ω-B 1A (13). Although clinical symptoms and treatment regimens are largely similar across TPA lineages and TEN, distinguishing them is important for understanding transmission dynamics and macrolide resistance patterns, potentially informing future public health strategies and interventions.

Conventional molecular surveillance methods, including enhanced Centers for Disease Control and Prevention typing (14), multi-locus sequence typing (MLST) (15), and whole-genome sequencing (7, 16), can discriminate between TPA lineages; however, they require time-consuming protocols, expensive equipment, and highly skilled laboratory technicians. Recently, whole-genome sequencing has been used for the detection of *tp0705* variants, which include three amino acid changes caused by four single-nucleotide polymorphisms (SNPs) at positions 506 (506A, 506T, and 506V), 625 (625M and 625V), and 708 (708G and 708S). These SNPs have the potential to discriminate between the major TPA lineages and TEN (8, 17). The development of rapid and easy-to-use typing methods to detect these variants is expected to contribute to epidemiological surveillance.

Loop-mediated isothermal amplification (LAMP), which is a nucleic acid amplification technique, is widely used in clinical diagnosis and disease surveillance because of its fast turnaround time, sensitivity, specificity, and low cost (18–21). Several SNP genotyping techniques using LAMP have been developed, and melting curve analysis combining fluorescence probes and LAMP is considered to be one of the most robust and reliable methods (22, 23). A quenching probe (QProbe) is a singly labeled oligonucleotide bearing a fluorescent dye (24). When a QProbe hybridizes with its target, electron transfer between the fluorophore and the guanine residue in the target sequence leads to fluorescence quenching. Any mismatches between the target sequence and QProbe results in a lower melting temperature during melting curve analysis. The combination of a QProbe and quantitative PCR and LAMP has been applied to SNP typing (23–26).

In this study, we developed and evaluated a novel LAMP method with QProbes for the rapid discrimination of TPA lineages and TEN in East Asia using clinical specimens.

## MATERIALS AND METHODS

### Clinical samples and DNA extraction

A total of 133 clinical samples consisting of genital swab (*n* = 105) and saliva (*n* = 28) samples collected from adult patients (≥18 years old) who were diagnosed with syphilis were used in this study. Some of the clinical samples had been analyzed by genotyping or whole-genome sequencing in a previous study (Table S1). Genital swabs were suspended in 1 mL phosphate-buffered saline. DNA was extracted from up to 200 µL of the specimens using a QIAamp DNA Mini Kit (Qiagen, Hilden, Germany) according to the manufacturer’s instructions. The extracted DNA was frozen at −80°C until analysis.

### Quantitative PCR

Quantitative PCR amplification of the *tp47* (*tp0574*) gene of TPA (27) was performed using TaqMan Fast Advanced Master Mix for qPCR (Applied Biosystems, Waltham, MA). Primer sequences are shown in Table S2. The final reaction volume was 20 µL, including 2 µL purified DNA sample. Thermal cycling consisted of 50°C for 2 min, 95°C for 20 s, and 45 cycles of denaturation at 95°C for 1 s and annealing/extension at 60°C for 20 s. A positive result was determined according to a Ct value of <45. Ten-fold serial dilutions of AMPLIRUN TREPONEMA DNA CONTROL (Vircell S.L., Granada, Spain) ranging from 2 to 2 × 10^3^ copies/reaction were used to estimate the *T. pallidum* DNA copy number in the specimens. The reactions were conducted in duplicate.

### MLST and multi-locus sequence analysis of *T. pallidum*

MLST of *T. pallidum* was performed according to Grillová et al. (15). Genes were amplified by nested PCR using KOD One (Toyobo, Osaka, Japan). The amplicons were analyzed by 1% agarose gel electrophoresis with ethidium bromide staining, and the PCR products were purified using a QIAquick Gel Extraction Kit (Qiagen). Direct Sanger sequencing was performed by Eurofins Genomics (Tokyo, Japan). The allelic profile was analyzed using the PubMLST BIGSdb database of *T. pallidum* (https://pubmlst.org/organisms/treponema-pallidum). Multi-locus sequence analysis of *tp0548* and *tp0856* to discriminate TPA and TEN was performed as previously described (12).

### Recombinant plasmids containing *tp0548* and *tp0705*

*tp0548* and *tp0705* were amplified from clinical specimens by PCR and cloned into the pCR4-TOPO vector (Thermo Fisher Scientific, Waltham, MA) using the In-Fusion cloning method. The pCR4-TOPO plasmid was digested with EcoRI, and the insertion sequences amplified by PCR from TPA lineages and TEN were inserted using an In-Fusion HD Cloning Kit (Takara Bio Inc., Shiga, Japan). Each recombinant plasmid was transformed into TOP10 competent *Escherichia coli*. Plasmid vectors were subsequently extracted using a QIAprep Spin Miniprep Kit (Qiagen) and sequenced by the Sanger method with M13 primers.

### Development of the LAMP genotyping assays

The oligonucleotide LAMP primer sets were designed using online LAMP primer design software (PrimerExplorer 5, https://primerexplorer.eiken.co.jp/; Eiken Chemical Co., Ltd., Tokyo, Japan). QProbes were synthesized by Tsukuba Oligo Service Co., Ltd. (Ibaragi, Japan). The QProbes were labeled with the fluorescent dye BODIPY FL at the cytosine residue of the 3′-end of the probe. Primer and QProbe sequences are shown in Table S2. The reactions were performed using Bst 3.0 DNA polymerase (New England Biolabs, Ipswich, MA). The final reaction volume was 25 µL, comprising 1.6 µM FIP and BIP primers, 0.8 µM LF and LB primers, 0.2 µM F3 and B3 primers, 2.5 µL of 10× Isothermal Amplification Buffer II, 6 mM MgSO_4_, 200 µM each dNTP, 5 µL DNA sample, 0.2 M betaine, 0.32 U/µL Bst 3.0 polymerase, 80 nM QProbe (for *tp0705*), and 1.25 µL EvaGreen Dye (for *tp0548*; Biotium, Inc., Fremont, CA). The LAMP reactions for *tp0705* and *tp0548* were carried out at 68°C for 80 and 20 min, respectively. After the LAMP reaction, melting curve analysis was conducted at 98°C for 3 min, 40°C for 1 min, and then from 40°C to 95°C at a ramp rate of 0.2 °C/s using a QuantStudio5 Dx Real-Time PCR System (Applied Biosystems).

### Phylogenetic analysis

Core genome-based phylogenetic trees were generated using PanACoTA (28) by comparing the deposited genomes in PubMLST and GenBank (Table S3 and S4). The genomes with a scaffold assembly (L90) exceeding 100 were discarded. All genomes were annotated with Prokka (29). The pan-genome was computed with a minimum sequence identity of 80%, and core genes were identified and aligned by PanACoTA with default parameters. The phylogenetic trees were constructed using the concatenated sequences of the core genes by IQ-TREE (30). Phylogenetic trees were generated using iTOL (https://itol.embl.de/).

### Statistical analysis

Continuous variables are expressed as the mean ± standard deviation or median (interquartile range) and were compared using a *t*-test or Wilcoxon rank-sum test for parametric or non-parametric data, respectively. All statistical analyses were performed using R (v.4.0.0; R Foundation for Statistical Computing, Vienna, Austria).

## RESULTS

### Phylogenetic analysis of *T. pallidum* in Asia

We focused on the genomic data of TPA and TEN isolates from Asia, performing an analysis to determine whether the *tp0705* gene could discriminate between the TPA lineages and TEN. Among the genomes available from PubMLST and National Center for Biotechnology Information, there were 100 TPA strains and 5 TEN strains isolated from Asia (Table S3 and S4). In the MLST allelic profile, SS14-East Asia of ST3 was the most prevalent with 66 strains, followed by SS14Ω-A of ST1 (*n* = 5), SS14Ω-A of ST2 (*n* = 5), Nichols of ST6 (*n* = 4), and SS14Ω-B 1A of ST11 (*n* = 2). The Nichols and SS14 genomes were used as the reference genomes for TPA, while the Bosnia A, C279, and C77 genomes were used for TEN. As a result of initial quality control, 13 TPA strains and 1 TEN strain were excluded from the analysis (Table S3 and S4). Because all ST11 strains were excluded by quality control (L90 >100), the genomes of the US and European isolates MD530xe, MD25x, MD24xe, OMI006, UW254B, and UW228B were added as references for ST11. Finally, 95 TPA genomes and 7 TEN genomes were used for subsequent analyses.

**Fig 1:**
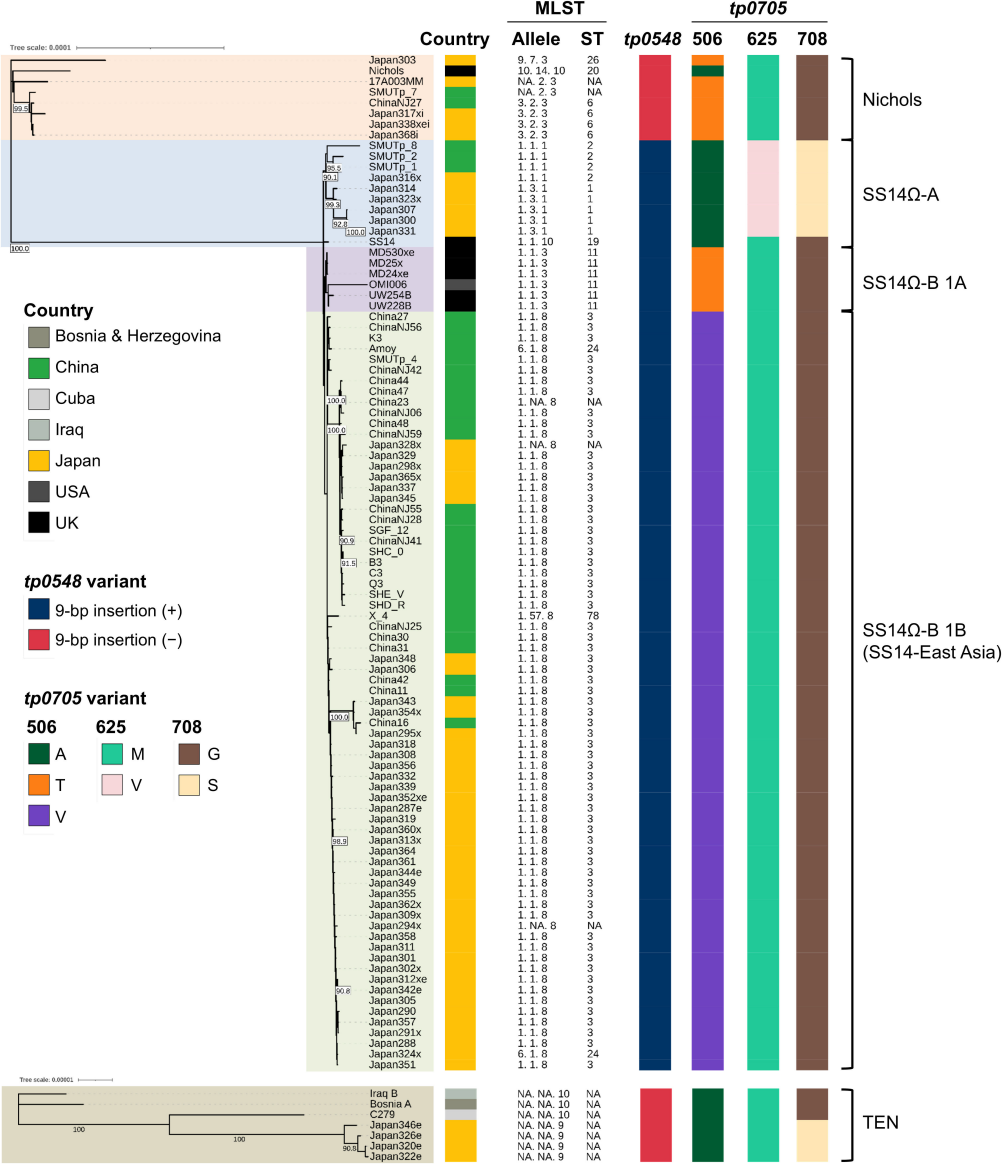
Phylogenetic trees of *Treponema pallidum* based on whole-genome sequencing. Phylogenetic trees were constructed using the concatenated sequences of 941 core genes for *Treponema pallidum* subsp. *pallidum* (*n* = 95) and 947 core genes for TEN) (*n* = 7) by IQ-TREE with ultrafast bootstrap approximation (UFBoot, *n* = 1,000). Bootstrap values >90% are indicated at the corresponding nodes. The scale bar shows branch length. MLST, multi-locus sequence typing; TEN, *Treponema pallidum* subsp. *endemicum*.

A maximum likelihood phylogenetic tree showed that the TPA isolates in Asia were classified into the Nichols, SS14Ω-A, SS14Ω-B 1A, and SS14-East Asia lineages (Fig. 1), consistent with previous reports (8, 17). The four TEN strains isolated from Japan belonged to the same clade and were phylogenetically distant from Iraq B and Bosnia A. In East Asia, amino acid analysis of *tp0705* revealed that the Nichols and SS14Ω-B 1A lineages had 506T, 625M, and 708G; the SS14Ω-A lineage had 506A, 625V, and 708S; and TEN had 506A, 625M, and 708S. Thus, we further investigated *tp0548* to discriminate the Nichols and SS14Ω-B 1A lineages. In *tp0548*, a 9 bp insertion sequence (3′-ACG-GTA-TGA-5′, positions 443–451) was found in the SS14 lineage but not in the Nichols lineage. By detecting the 506, 625, and 708 amino acid variants of *tp0705* along with the 9 bp insertion in *tp0548*, the TPA lineages (Nichols, SS14Ω-A, SS14Ω-B 1A, and SS14-East Asia) and TEN could be distinguished in Asian samples.

### LAMP primer and QProbe design

**Fig 2:**
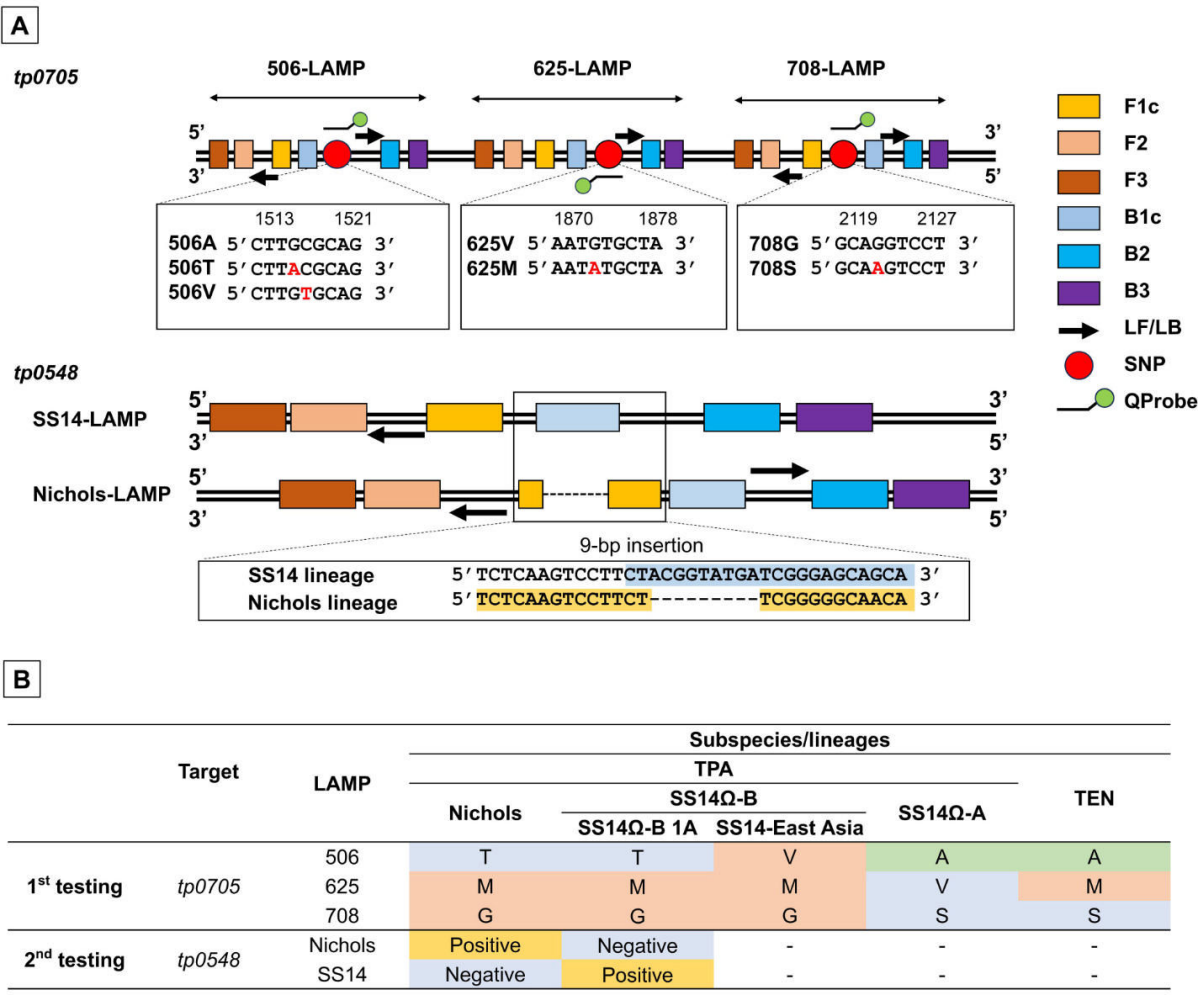
Loop-mediated isothermal amplification (LAMP) genotyping for *Treponema pallidum* lineages and subspecies. (**A**) Primer and probe design. For *tp0705*, LAMP primers were designed to amplify four single-nucleotide polymorphisms (SNPs) that cause amino acid substitutions at positions 506 (A, T, and V), 625 (V and M), and 708 (G and S). The nucleotide sequences of the SNPs are highlighted in red. Quenching probes (QProbes) were designed to be complementary to the 506A (sense), 625V (antisense), and 708G (sense) alleles, respectively. For *tp0548*, LAMP primers were designed to be able to distinguish the SS14 and Nichols-like sequences. The primer set was designed such that the B1c and F1c primers are located across the 9 bp deletion junction in SS14-LAMP and Nichols-LAMP, respectively. (**B**) Genotyping flow of the LAMP assays. Genotyping of *tp0705* by 506-, 625-, and 708-LAMP is used for the first round of testing. If 506T, 625M, and 708G are detected, an additional LAMP assay targeting *tp0548* will be implemented to determine whether Nichols or SS14Ω-B 1A is present. TEN, *Treponema pallidum* subsp. *endemicum*; TPA, *Treponema pallidum* subsp. *pallidum*.

We designed a LAMP method to detect *tp0705* variants and specifically target the 9 bp insertion in *tp0548*. LAMP primer and probe positions and sequences are shown in Fig. 2A and Table S2. The genotyping scheme is shown in Fig. 2B. First, the 506, 625, and 708 variants of *tp0705* were determined. If 506T, 625M, and 708G, which are shared by Nichols and SS14Ω-B 1A, were detected, an additional analysis of the *tp0548* region was conducted. For *tp0705*, LAMP primer sets were designed to amplify the areas containing the 506, 625, and 708 amino acid positions (506-LAMP, 625-LAMP, and 708-LAMP, respectively). QProbes that bound to each region containing the 506A, 625V, and 708G alleles were also designed. The cytosine base at the 3′-end of the QProbes was labeled with BODIPY FL. A loop primer set (LF and LB), which accelerates the LAMP reaction, was also designed for each primer set. For 625-LAMP, the designed LB primer overlapped with the QProbe-binding region. Thus, we added three artificially mutated nucleic acids in the LB to avoid the effect of the amplified DNA segment driven from the LB for melting curve analysis (Table S2). For *tp0548*, two LAMP primer sets, SS14-LAMP and Nichols-LAMP, were designed to detect the SS14-like and Nichols-like allele, respectively. A primer set in which F1c was located across the 9 bp insertion-junction in Nichols-LAMP and in which B1c was located on the 9 bp insertion in SS14-LAMP was designed (Fig. 2A).

### Basic performance of the LAMP assays

**Fig 3:**
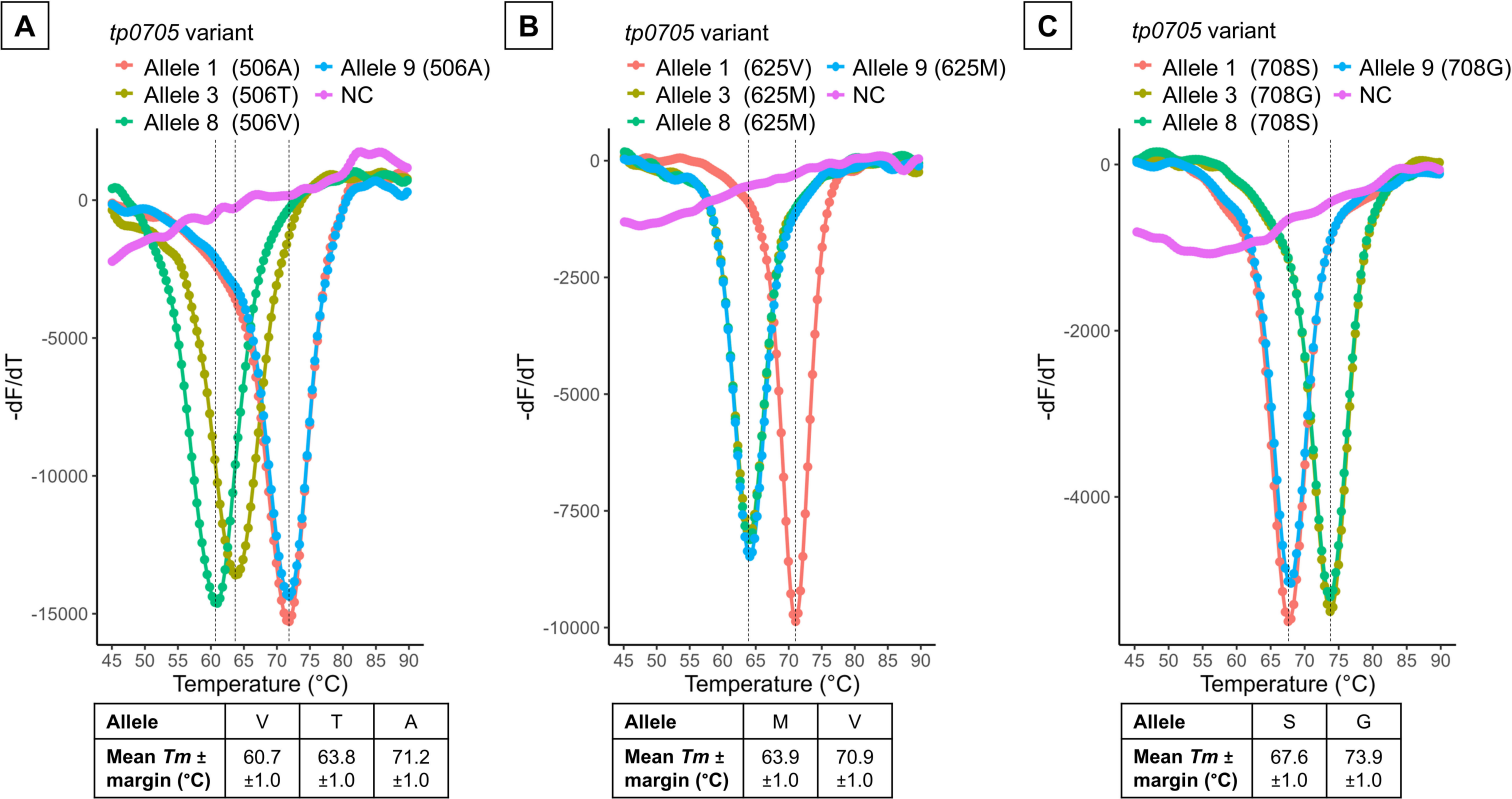
Loop-mediated isothermal amplification (LAMP) assays for *tp0705*. Melting peak calculation by derivative plotting -dF/dT versus temperature in 506-LAMP (**A**), 625-LAMP (**B**), and 708-LAMP (**C**). The average melting temperature (*Tm*) of each allele was calculated using eight replicates, each containing recombinant plasmids at a concentration of 2.0 × 10^3^ copies/tube, and the results are shown below. NC, negative control (distilled water).

**Fig 4:**
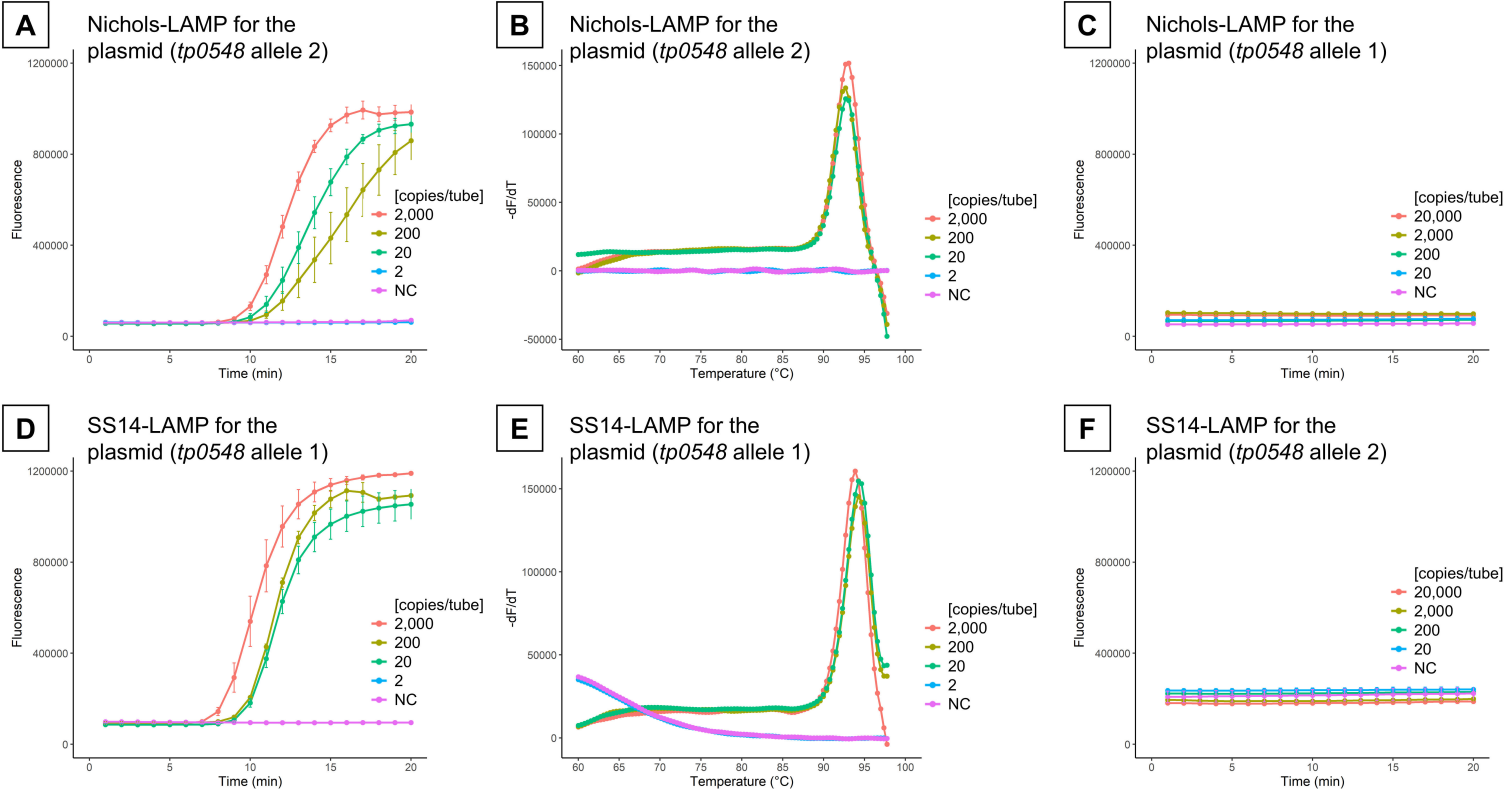
Loop-mediated isothermal amplification (LAMP) assays for *tp0548*. The limit of detection for Nichols-LAMP and SS14-LAMP (**A and D**) and subsequent melting curve analysis (**B and E**). Specificity of the Nichols-LAMP (**C**) and SS14-LAMP (**F**) assays. Samples of each concentration were tested in the fourth replicate. Error bars indicate standard error intervals (**A and D**). NC, negative control (distilled water).

Four plasmids containing the *tp0705* alleles of MLST (allele 1: 506A, 625V, and 708S; allele 3: 506T, 625M, and 708G; allele 8: 506V, 625M, and 708G; and allele 9: 506A, 625M, and 708S) were amplified by 506-, 625-, and 708-LAMP at 68°C for 80 min. As expected, melting curve analysis indicated a single allele, and the QProbes had a lower affinity for the mismatch sequence, resulting in a decreased *Tm* (Fig. 3). In addition, the *Tm* margin for each variant was set to ±1°C, taking into account potential variations due to experimental error and contaminants present in the DNA extracts. The detection limit of the LAMP assays was 20 copies of plasmid per reaction (Fig. S1). For *tp0548*, SS14-LAMP and Nichols-LAMP detected 20 copies of plasmid containing *tp0548* MLST allele 1 (SS14 lineage, 9 bp insertion) and allele 2 (Nichols lineage, non-insertion) per tube at 68°C for 20 min, respectively (Fig. 4). Melting curve analysis following the LAMP reaction produced a single peak at 92.46°C ± 0.13°C and 93.71°C ± 0.21°C for SS14-LAMP and Nichols-LAMP, respectively, suggesting specific amplification had occurred during the reactions. There was no cross-reaction of Nichols-LAMP and SS14-LAMP for up to 2.0 × 10^4^ copies of plasmid containing *tp0548* MLST allele 2 and 1, respectively (Fig. 4).

### LAMP assays using clinical specimens

Next, we tested the performance of the LAMP assays with clinical specimens. The lineages and subspecies of *T. pallidum* in clinical specimens were determined by whole-genome sequencing when such data were available (Table S1). For samples for which this approach was not feasible, MLST and MLSA were used instead. The analysis revealed that the most common *T. pallidum* lineage and subspecies was the SS14-East Asia lineage (*n* = 110), followed by the Nichols lineage (*n* = 9), SS14Ω-A lineage (*n* = 7), TEN (*n* = 5), and SS14Ω-B 1A lineage (*n* = 2). Of the 133 samples, the LAMP assays fully typed the allelic profile of *tp0705* in 129 (97.0%) samples, and 1 (0.7%) and 3 (2.2%) samples were partially or not typed, respectively (Table 1 and Table S1). According to the results of the LAMP assays for *tp0705*, 7 samples were the 506A, 625V, and 708S variants; 106 samples were the 506V, 625M, and 708G variants; 11 samples were the 506T, 625M, and 708G variants; and 5 samples were the 506A, 625M, and 708S variants. The 506T, 625M, and 708G variant samples were subsequently analyzed by the LAMP assays for *tp0548* to determine the Nichols and SS14 lineages. Of the 11 samples, 9 and 2 were positive in Nichols-LAMP and SS14-LAMP, respectively. Finally, the LAMP assays classified the *T. pallidum* lineages and subspecies as Nichols (*n* = 9), SS14Ω-A (*n* = 7), SS14Ω-B 1A (*n* = 2), SS14-East Asia (*n* = 106), and TEN (*n* = 5). The genotyping results of the LAMP assays were completely consistent with those of MLST.

**TABLE 1:** Genotyping results of the LAMP assays for clinical specimens (*n* = 133)^*a*^

Lineages and subspecies	Whole-genome sequencing and Sanger sequencing		LAMP					
	tp0705 variant	tp0548 variant	No. of tp0705 variant (%)				No. of tp0548 variant (%)	
			506A 625V 708S	506V 625M 708G	506T 625M 708G	506A 625M 708S	Nichols	SS14
SS14Ω-A (*n* = 7)	506A 625V 708S	SS14	7/7 (100)	–^*c*^	–		–	–
SS14-East Asia (*n* = 110)	506V 625M 708G	SS14	–	106/110^*b*^ (96.4)	–		–	–
Nichols (*n* = 9)	506T 625M 708G	Nichols	–	–	9/9 (100)		9/9 (100)	–
SS14Ω-B 1A(*n* = 2)	506T 625M 708G	SS14	–	–	2/2 (100)		–	2/2 (100)
TEN (*n* = 5)	506A 625M 708S	Nichols	–	–	–	5/5 (100)	–	–

^*a*^ LAMP, loop-mediated isothermal amplification; TEN, *Treponema pallidum* subsp. *endemicum*.

^*b*^ Three specimens and one specimen were untypable or partially typed (only 625-LAMP was typable) by LAMP assays, respectively. TEN, *Treponema pallidum* subsp. *endemicum*.

^*c*^ “–” indicates that the data are not available.

The *T. pallidum* DNA copy number distribution and LAMP results for clinical specimens are shown in Fig. S2. The *T. pallidum* DNA copy number was significantly lower in the clinical samples that were partially or not typed for the *tp0705* allele by the LAMP assays than in the fully typed samples (median 23.9 copies/tube [interquartile range, 15.41–24.9] versus median 1,037.9 copies/tube [interquartile range, 320.1–3462.7]; *P* < 0.001).

## DISCUSSION

In this study, we developed and evaluated a novel method to discriminate *T. pallidum* lineages and subspecies using LAMP assays. Past reports have indicated that *T. pallidum* lineages and subspecies can be differentiated by *tp0705* allele typing (8, 17). Despite their low frequency, several strains belonging to the SS14Ω-B 1A lineage have the MLST allele 3 (506T, 625M, and 708G), similar to the Nichols lineage, and have been reported in Asia. Thus, we added *tp0548* typing targeting a 9 bp insertion to distinguish between the SS14 and Nichols lineages. By combining *tp0705* and *tp0548* typing, we have shown that it is possible to differentiate all reported *T. pallidum* lineages and subspecies in Japan.

According to previous reports in Japan, the SS14-East Asia lineage is predominant among heterosexual patients and men who have sex with men (MSM) patients (17). Nichols, SS14Ω-A, and TEN are detected in MSM patients, while they are rare in heterosexual patients (8, 12, 17, 31). It is suggested that different lineages and subspecies are circulating in the heterosexual and MSM populations. However, due to the small sample size and limited geographic area of epidemiological studies, it cannot be concluded that this truly reflects the overall circulation of *T. pallidum* in Japan. By combining LAMP testing with information on gender and sexual orientation, it will be possible to investigate the detailed aspects of strain circulation in Japan.

LAMP-based SNP detection methods can be categorized broadly into two approaches. The first approach involves the use of specialized primer design that increases specificity, i.e., ARMS-LAMP (32) and SNP-LAMP (33), or the use of a blocking probe with a complementary SNP sequence, i.e., PNA-LNA-LAMP (34) and PNA-LAMP (35), to perform allele-specific amplification. This approach can determine the results visually; thus, it has been favored for on-site clinical diagnostics. However, a major disadvantage of these methods is the risk of false-positive results, difficulty in primer design, and reduced sensitivity due to the influence of blocking primers. The second approach uses a fluorescent DNA probe and non-allele-specific amplification using quantitative PCR (22, 23), which has advantages such as high flexibility in primer design as well as high sensitivity and specificity. This approach is suitable for epidemiological studies that require handling multiple samples and accurate genotyping, but it does not necessitate on-site diagnostics. In the present study, we leveraged LAMP and QProbes for the specific discrimination of up to three different alleles. The robustness of our method was high, and the results of SNP typing were completely consistent with those of Sanger sequencing. The sensitivity of our assays was equivalent to previously reported LAMP assays for diagnosing syphilis (36).

A major limitation of our method is its low discrimination power. Our method is not intended to replace MLST or whole-genome sequencing in the molecular surveillance of *T. pallidum*. However, it can simplify the differentiation of *T. pallidum* lineages and subspecies, leading to a reduction of the cost and time required for molecular surveillance in East Asian countries. In addition, it offers practical advantages, including rapid and low-cost testing that can be performed in settings without access to sequencing facilities, including local clinics or laboratories. Notably, the estimated cost per sample using our method (USD 4.11–6.85, Table S5) is considerably lower than that of MLST (USD 16 per sample for sequencing three genes from both strands outsourced), highlighting its cost-effectiveness. By choosing the appropriate method based on the situation, we expect to accelerate domestic epidemiological investigations and enable rapid and effective planning of infection control measures for syphilis.

In conclusion, our LAMP assays for *tp0705* and *tp0548* genotyping can discriminate *T. pallidum* subspecies and lineages that are circulating in East Asia with rapidity and ease. This method will contribute to the epidemiological surveillance of *T. pallidum* in East Asia.

## Data Availability

The data sets analyzed during the present study are available from the corresponding author on reasonable request.

## SUPPLEMENTAL MATERIAL

Fig. S1 spectrum.01565-25-s0001.tif Loop-mediated isothermal amplification (LAMP) assays for *tp0705* for a serial dilution of each recombinant plasmid.

Fig. S2 spectrum.01565-25-s0002.tif *Treponema pallidum* DNA copy number.

Supplemental tables spectrum.01565-25-s0003.xlsx Tables S1 to S5.

ASM does not own the copyrights to Supplemental Material that may be linked to, or accessed through, an article. The authors have granted ASM a non-exclusive, world-wide license to publish the Supplemental Material files. Please contact the corresponding author directly for reuse.
