# Supplementary figures and images for: Loop-mediated isothermal amplification assays for the rapid discrimination of Treponema pallidum lineages and subspecies
Source: Microbiol Spectr. 2025 Oct 30;13(12):e01565-25. doi: 10.1128/spectrum.01565-25 (PMC12671098; doi:10.1128/spectrum.01565-25)

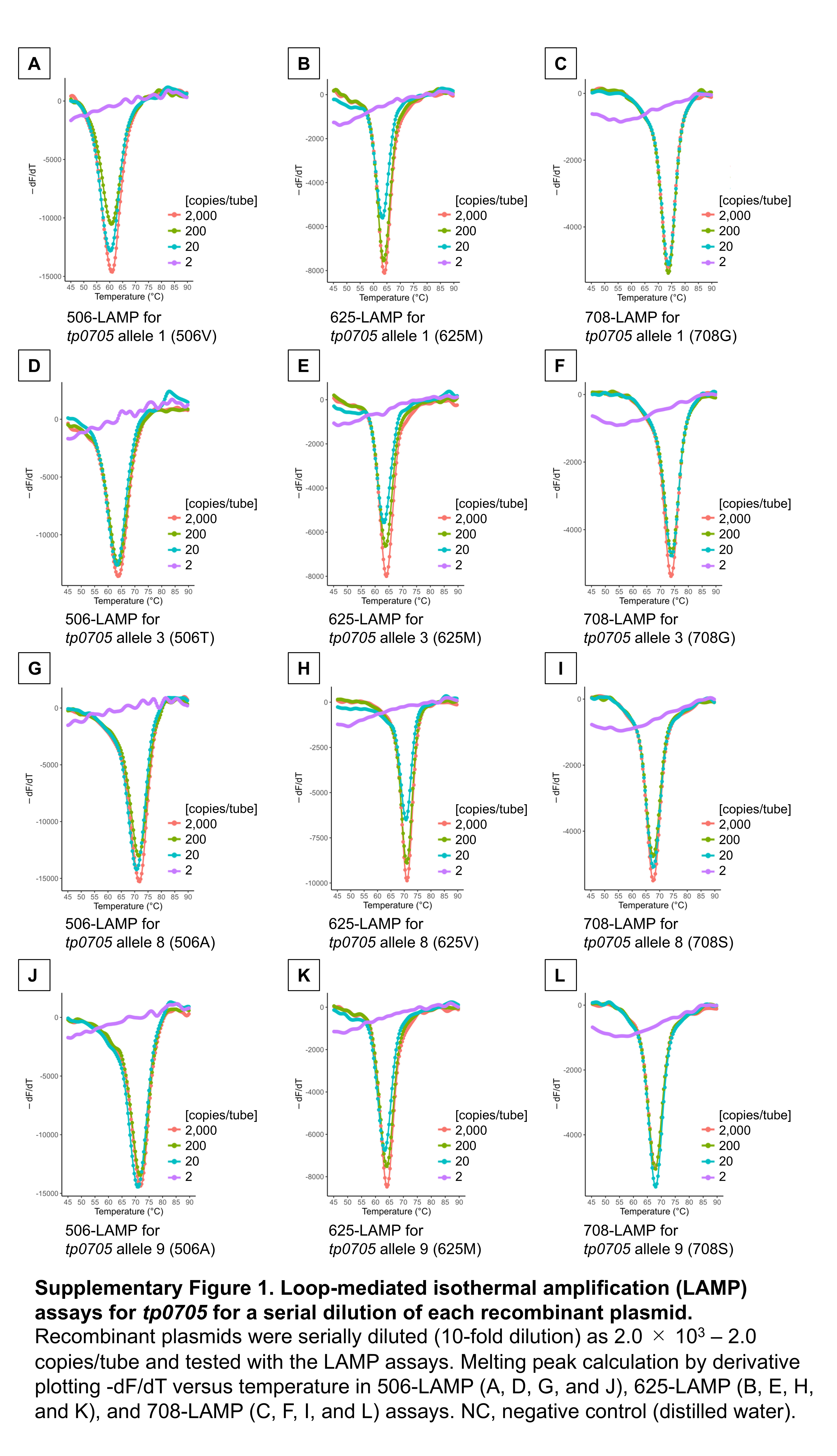

Supplement: Fig. S1 — Loop-mediated isothermal amplification (LAMP) assays for tp0705 for a serial dilution of each recombinant plasmid. [file spectrum.01565-25-s0001.tif]

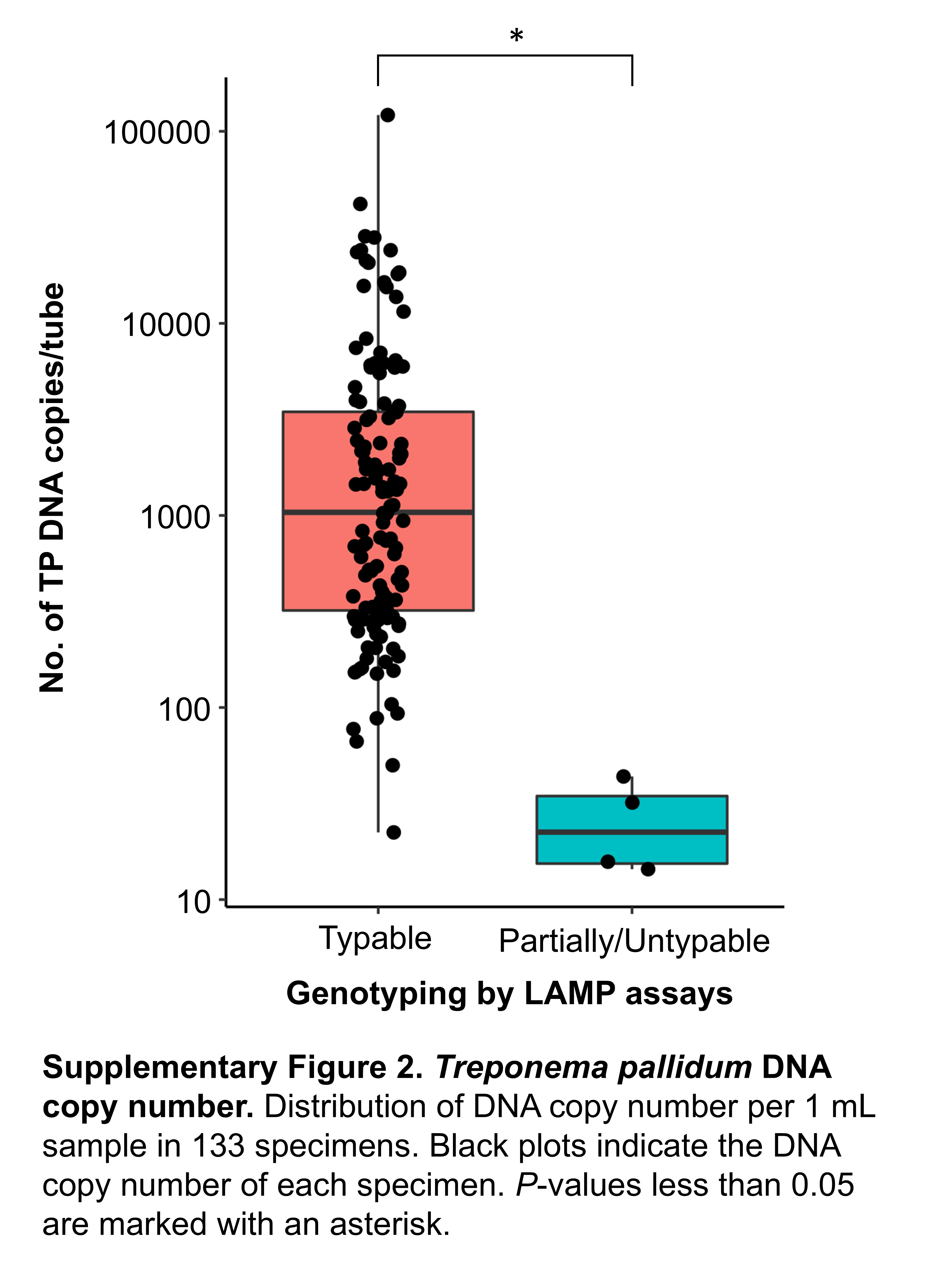

Supplement: Fig. S2 — Treponema pallidum DNA copy number. [file spectrum.01565-25-s0002.tif]
